# Quantification of AICAR and study of metabolic markers after administration†

**DOI:** 10.1039/d4ra02878c

**Published:** 2024-06-13

**Authors:** Jingyi Fan, Yirang Wang, Yue Zhuo, Siyan Xu, Wanggeng Zhou, Bing Liu

**Affiliations:** a Shanghai University of Sport Changhai Road 399 Shanghai 200438 P. R. China liubing2019@sus.edu.cn; b Xiamen Medical College 1999 Guankou Road, Jimei District Xiamen Fujian 361023 P. R. China

## Abstract

*Objectives*: AICAR (5-amino-4-imidazolecarboxyamide ribonucleoside) was reported as the first pharmacological AMPK (adenosine 5′-monophosphate (AMP)-activated protein kinase) activator, and it has been confirmed to exhibit a significant endurance enhancement effect and prohibited for doping by the World Anti-Doping Agency. Due to the fact that the human body can produce such substances, in order to ensure fairness in sports competition, methods for rapid detection and multi-type identification of AICAR drugs taken orally should be established. *Methods*: to assess AICAR levels, a new rapid, sensitive, efficient, and selective method was reported for the quantitative detection of AICAR in urine using LC-MS/MS. The method was validated for quantitative purposes based on the elemental selectivity, intra- (1.0–15.6%) and inter-day precision (1.3–16.3%), accuracy (99.9–112.8%), matrix effects (88.9–103.6%), recovery (87.4–106.5%), and stability at four different concentrations. The calibration curve was linear over a wide concentration range of 10–10,000 ng mL^−1^ with a high coefficient of determination (*R*^2^ > 0.998). The limit of detection (LOD) and limit of quantification (LOQ) for the experiment were determined to be 1 and 10 ng mL^−1^, respectively. Simultaneously, metabolomics analysis was used to obtain the metabolic fingerprint of different populations and biomarkers to distinguish administration cases through partial least squares discriminant analysis (PLS-DA) and a receiver operating characteristic (ROC) curve. *Results*: the method enables easy quantitation for LC-MS/MS analysis with the best recovery yield maintained, and the method was applied to 122 Asian biological samples with an average concentration of 1310.5 ± 1031.4 ng mL^−1^. Through drug metabolism research, 734 and 294 variables were extracted for data analysis respectively in the positive and negative ion modes, and more than 100 metabolites with significant up- and down-regulation were found after the test. *Conclusions*: this research developed a fast, precise, effective, and specific approach for the qualitative and quantitative identification of AICAR in urine. Meanwhile, administration metabolism studies found that there were significant changes in AICAR levels and other compounds, such as PC types PC(18:1/16:0), PC(16:0/18:0), and PC(16:0/16:0), PE types PE(18:0/20:4), and LPE-type 18:1, which could better distinguish samples before and after AICAR administration. The analysis provides a multi-perspective reference for WADA to determine a positive criterion.

## Abstract 2

AICAR was reported as the first pharmacological AMPK activator, and it has been confirmed to exhibit a significant endurance enhancement effect and prohibited for doping by the World Anti-Doping Agency.

## Graphical abstract

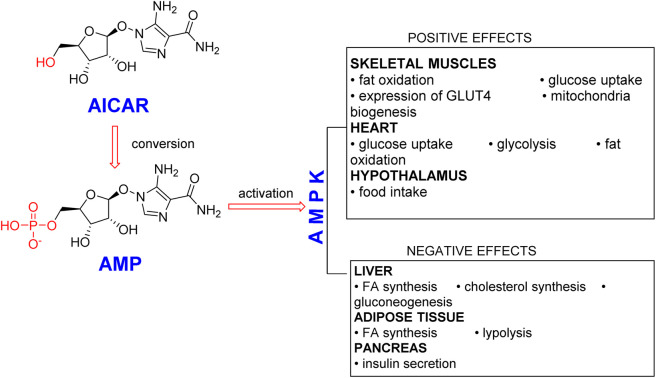

## 1 Introduction

**Fig. 1:**
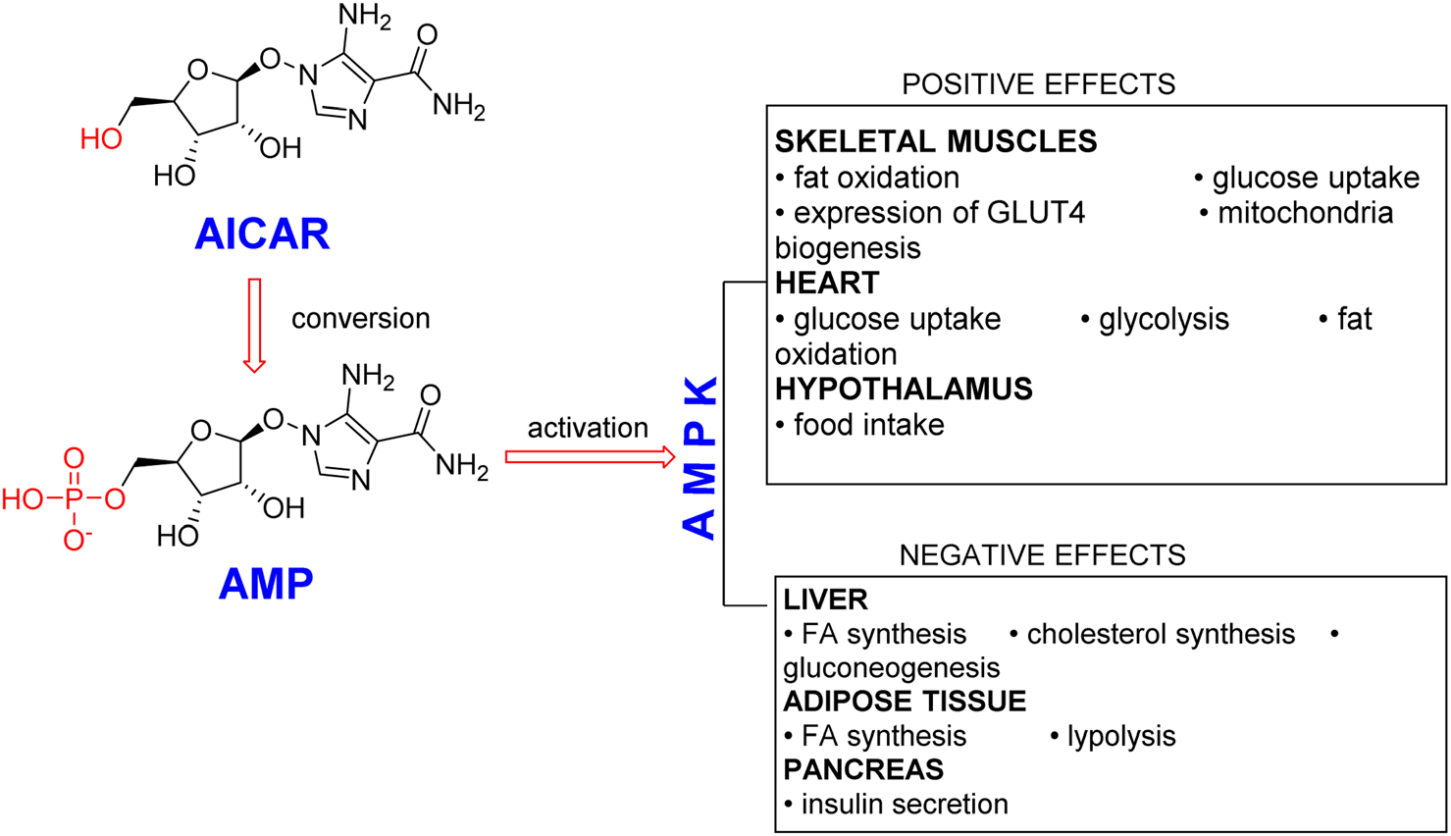
Pharmacological role of AICAR as an AMPK activator.

AICAR is the first reported pharmacological AMPK activator, and it has received extensive clinical attention^1,2^ (Fig. 1). By down-regulating the expression of adipogenic factors and activating PGC1α expression *in vitro*, AICAR can inhibit adipocyte differentiation and decrease the content of adipose tissue in mice with diet-induced obesity.^3–5^ In addition, AICAR has been reported to produce an extra benefit in preventing endoplasmic reticulum stress and protein unfolding responses,^6^ and the effect of AICAR on both cultured HL-1 cardiomyocytes was studied to explore the possibility of preventing cardiomyocyte injury.^7^ AICAR has also been used as an experimental tool to activate AMPK *in vitro* and *in vivo*.^8^ AICAR transforms to ZMP (AICAR monophosphate) by adenosine kinase inside a cell, and ZMP can act as an AMP analogue to activate AMPK in an LKB1-dependent manner.^9^

Once ZMP is formed, NTPs (ribonucleoside triphosphates) produced *de novo* can be employed for the synthesis of certain RNA.^10^ Various studies on the mechanism confirmed that the transcription of such genes has a significant effect on the endurance enhancement by increasing the activity of transcriptional regulators at a genetic level and enhancing the expression of genes in skeletal muscle.^11,12^ As a result, doping with the substance AICAR was prohibited by the World Anti-Doping Agency (WADA)^13^ in 2009, and its misuse was classified *via* metabolic regulators after three years.

**Fig. 2:**
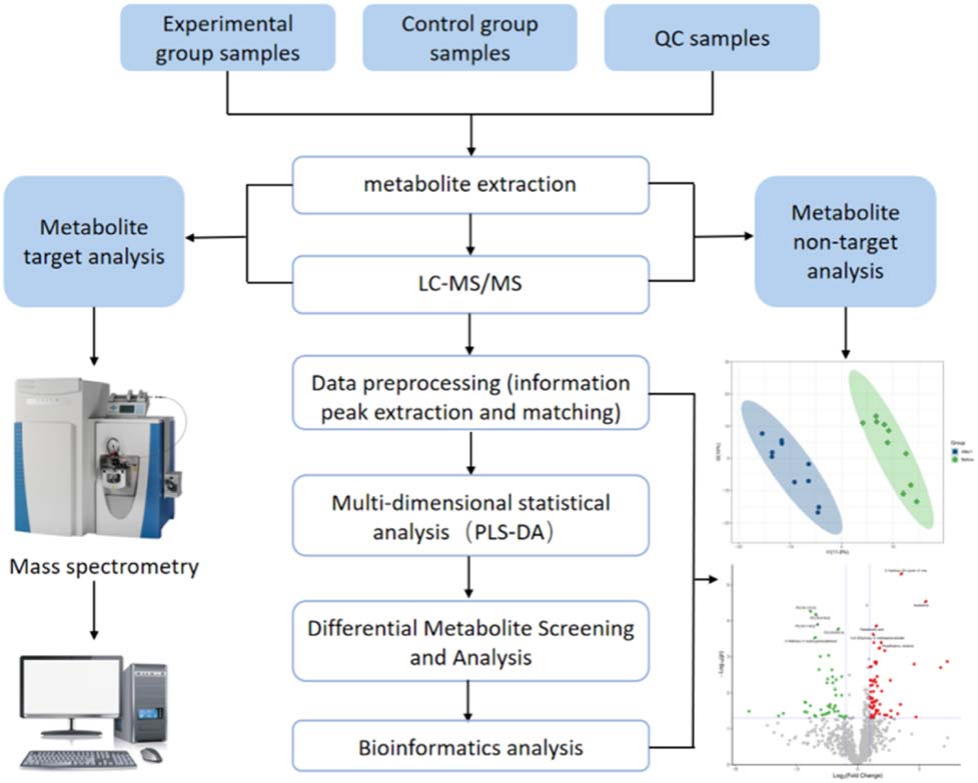
Workflow of the analysis process.

Recently, with the development of detection methods in the mass spectrometry industry,^14^ some cases have been reported for the qualitative and quantitative analyses of AICAR contents *in vivo*.^15–17^ Thevis' group has previously reported a detection method in which AICAR is directly dissolved and injected, and the limit of quantification (LOQ) was 100 ng mL^−1^ in 2010.^15^ Subsequently, the group proposed a new concept of “dilute-and-shoot” using a liquid chromatography (LC)-mass spectrometry (MS)/MS multi-target method for the semi-quantitation of prohibited compounds containing AICAR, which had a limit of detection (LOD) of 30 ng mL^−1^.^16^ Direct detection and analysis of urine samples brings convenience to testing personnel, but it also causes rapid contamination problems of the ion sources and analysis column due to the accumulation of inorganic salts and organic metabolites in urine. To solve the matrix interference problem, Dmitrieva *et al.*^17^ published a quantitative detection approach of AICAR through improved solid-phase extraction preprocessing, and the limit of quantification was 50 ng mL^−1^ with an error of quantification of less than 15% evaluated by the calibration curve. In addition to the above-mentioned matrix effects in the detection methods, current quantitative methods also ignore the concentration of the AICAR bound to enzymes *in vivo*.^9,18^ Due to the fact that the human body can produce such substances and there are also significant individual concentration differences, there is no public document that clearly indicates whether an athlete has taken the drug based on their concentration. To ensure fairness in sports competition, methods for rapid detection and multi-type identification of AICAR drugs taken orally should be established (Fig. 2).

## 2 Materials and methods

### 2.1. Materials

To diminish the potential matrix effects, an ^15^N, ^13^C_2_-labelled AICAR internal standard (AICAR-IS) was employed to obtain the correct quantitative results. AICAR and AICAR-IS were purchased from TRC. All the high-performance liquid chromatography (HPLC)-grade solvents and ammonium formate were obtained from Merck. β-Glucuronidase (*E. coli*) and formic acid were purchased from Sigma-Aldrich. Phosphates were purchased from Adamas and Energy Chemical. A Kinetex F5 column was employed (2.1 mm × 100 mm, 2.6 μm, Phenomenex, USA).

A 1290 infinity high-performance liquid chromatograph (Agilent Technologies, USA) and QTRAP 6500+ mass spectrometer were used. The mass data were collected in the MRM mode with ESI polarity switching.

### 2.2. Instrumentation conditions

#### 2.2.1. Determination and quantification conditions

Liquid chromatography was performed at 40 °C column temperature and 0.3 mL min^−1^ flow rate with the mobile phase of water containing 0.1% formic acid, and an injection volume of 2 μL with a total run time of 3 min. The retention time (RT) of AICAR was 1.69 min under the set conditions.

**Fig. 3:**
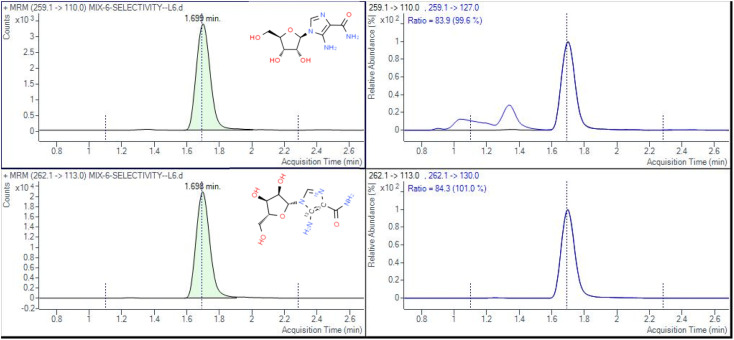
Multiple reaction mode spectra of AICAR (upper) and AICAR-IS (lower).

**Fig. 4:**
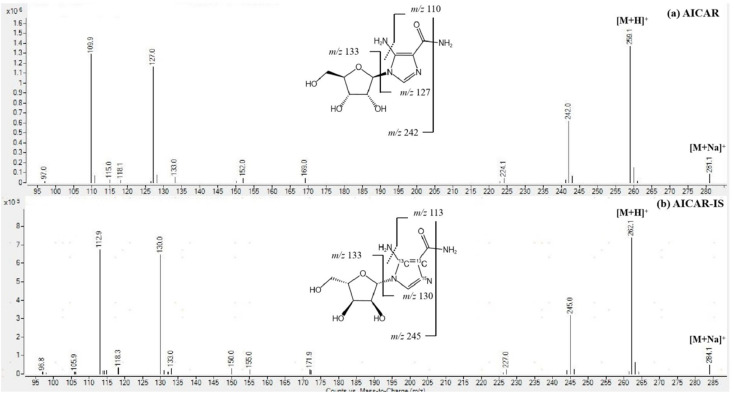
MS/MS spectrum of (a) AICAR and (b) AICAR-IS, all employing the respective protonated molecules [M + H] as precursor ions.

The determination of AICAR and AICAR-IS was conducted in the positive ion mode (ESI) using a QTRAP 6500+ mass spectrometer. This analytical approach allowed for accurate and reliable measurements of these compounds. In addition, N_2_ was utilized as the nebulizing gas (3 L min^−1^) and drying gas (15 L min^−1^), and the following gas configuration was used: collision gas (CAD) (N_2_) pressure = medium, curtain gas (N_2_) pressure = 35 a.u., ion spray voltage = 5.2 kV, and temperature = 550 °C. The mass scanning range was *m*/*z* = 100–500 in full data storage mode. The MRM transition ions identified for AICAR were at *m*/*z* = 259.1 > 110.0 and *m*/*z* = 259.1 > 127.0. Additionally, the transition ions for AICAR-IS were found at *m*/*z* = 262.1 > 113.0 and *m*/*z* = 262.1 > 130.0. Under the current LC-MS conditions, there are several notable characteristics in the chromatogram. (1) The retention time window differences are within 0.1 min (Fig. 3). (2) Both standard solutions at the same concentration have similar peak shapes and good peak intensities on the chromatograms. Thus, it was speculated that these characteristics will also be present in the labeled and unlabeled metabolites (Fig. 3). (3) AICAR and AICAR-IS have similar fragmentation patterns in mass spectrometry (Fig. 4).These specific mass-to-charge ratios provide key information for the analysis and identification of AICAR and its internal standard AICAR-IS in this study, providing strong support for subsequent verification of methodological indicators such as selectivity, LOD and LOQ, highlighting the importance of the methods used.

#### 2.2.2. Metabolomics profiling conditions

For liquid chromatography (LC) separation, samples were analyzed in the binary gradient mode using a ACQUITY UPLC® HSS T3 column (2.1 × 100 mm, 1.8 μm) (Waters, Milford, MA, USA). The flow rate was 0.3 mL min^−1^ and the mobile phase contained 0.1% FA in water (A) and 100% acetonitrile (ACN) (B). The gradient was 0% buffer B for 2 min, linearly increased to 48% in 4 min and up to 100% in 4 min, maintained for 2 min, and then decreased to 0% buffer B in 0.1 min, with 3 min re-equilibration period employed.

The samples were analyzed using UPLC-Q-Exactive Plus MS technology combined with data-dependent acquisition to perform full-spectrum analysis, obtaining both primary and secondary mass spectrometry data. UPLC-Q-Exactive Plus MS analysis was performed using a quadrupole-Orbitrap mass spectrometer (Thermo Scientific, Q-Exactive Plus) equipped with an LC-30AD ultra-high-performance liquid chromatography system (Shimadzu, Kyoto, Japan) in both positive and negative ion modes. The electrospray ionization (ESI) with positive and negative modes was applied for MS data acquisition separately. The HESI source conditions were set as follows: spray voltage = 3.8 kV (positive) and 3.2 kV (negative); capillary temperature = 320 °C; sheath gas (nitrogen) flow = 30 arb (arbitrary units); Aux gas flow = 5 arb; probe heater temp = 350 °C; S-lens RF level = 50. The instrument was set to acquire over the *m*/*z* range 70–1050 Da for full MS. The full MS scans were acquired at a resolution of 70 000 at *m*/*z* 200, and 17 500 at *m*/*z* 200 for MS/MS scan. The maximum injection time was set to 100 ms for MS and 50 ms for MS/MS. The isolation window for MS2 was set to 2 *m*/*z* and the normalized collision energy (stepped) was set as 20, 30 and 40 for fragmentation.

The autosampler and column temperatures were set to 4 °C and 40 °C, respectively, with an injection volume of 4 μL. The samples were run using a stratified method, with samples from different subjects placed in a random order. After every 10–12 urine samples, a QC sample and a blank vial were run.

### 2.3. Standard solution, calibration standard, and quality controls

Appropriate standards of AICAR and AICAR-IS were accurately weighed in bottles, after which they were dissolved in methanol to obtain a standard stock solution of 1.00 mg mL^−1^. They were then diluted into standard working solutions at concentrations of 100 μg mL^−1^ and 2 μg mL^−1^ and stored at −20 °C.

The standard working solution was diluted with H_2_O to 1 ng mL^−1^, 10 ng mL^−1^, 100 ng mL^−1^, 100 ng mL^−1^, 500 ng mL^−1^, 1000 ng mL^−1^, 2000 ng mL^−1^, 5000 ng mL^−1^, and 10 000 ng mL^−1^ concentrations.

QC samples were prepared by spiking human urine with working solutions and stock solutions reaching concentrations at LLOQ (10 ng mL^−1^), LQC (100 ng mL^−1^), MQC (1000 ng mL^−1^) and HQC (5000 ng mL^−1^) levels.

### 2.4. Sample pre-treatment

To start the quantification experiment, a total of 100 μL of internal standard (2 μg mL^−1^, solvent PBS), 10 μL of MeOH, and 10 μL of enzyme (β-glucuronidase) were carefully combined with 200 μL of urine in a thorough mixing process. The solution was enzymatically hydrolyzed at 55 °C in a water bath for 2 h. After the enzymatic solution was cooled to room temperature, 200 μL of the mixed solution was added to 200 μL of MeOH. The solution was shaken at 2500 rpm for 10 s, followed by centrifugation at a temperature of 4 °C and a rotation speed of 13 000 rpm for 15 min. Then, 200 μL supernatant was mixed with 800 μL of water, and the resulting sample was stored for future testing. After each batch of samples were tested, the column was rinsed with an organic/water mixed mobile phase.

For metabolomics analysis after administration of AICAR. All collected urine samples were stored at −80 °C until analysis. Prior to analysis, the samples were moved out and thawed at 4 °C. Then 100 μL of urine sample and 400 μL of pre-cooled pure methanol were taken, vortex mixed, sonicated in an ice bath for 20 minutes, allowed to stand at −20 °C for 1 hour, and centrifuged at 16 000 g, at 4 °C for 20 minutes, and the supernatant was collected and evaporated in a high-speed vacuum concentrator. Before loading, 100 μL of methanol–water solution (1 : 1, v/v) was added for reconstitution and then centrifuged at 20 000*g*, at 4 °C for 5 minutes. A blank was prepared using a 50% methanol–water solution. All samples were mixed equally to prepare a quality control (QC) sample to ensure the stability of the analysis conditions and process it using the above-mentioned method.

### 2.5. Method validation

In order to ensure accurate results for the detection of AICAR in urine samples, it was necessary to validate the method for quantification purposes. The validation parameters were the selectivity, carry-over, linearity range, precision (intra/inter-day), accuracy, LOD, LOQ, matrix effect, recovery, and stability.

#### 2.5.1. Selectivity

For selectivity testing, the standard solutions were tested for possible chromatogram interference and the ion chromatograms represent good selectivity. AICAR in different urine samples was identified by comparing its retention time with that of pure standard. Meanwhile, different urine samples were also collected and spiked with the internal standard, and consistent results were obtained by comparison of chromatograms.

#### 2.5.2. Carry-over effect

Carry-over was assessed by injecting blank samples after the analysis of a high-concentration standard (10 000 ng mL^−1^) of the calibration curve during the validation.

#### 2.5.3. Linearity range

As described above, the standard working solution was prepared (10 to 10 000 ng mL^−1^). The blank matrix was fortified with increasing amounts of target analytes. With the concentration *X* (ng mL^−1^) of each analyte as the abscissa and the ratio *Y* of the peak area of the target substance to the peak area of the internal standard as the ordinate, a standard curve was drawn, and the regression equation and correlation coefficient were obtained.

#### 2.5.4. Accuracy and precision

The accuracy and precision were tested at four concentration levels (LLOQ, LQC, MQC and HQC) with four repetitions on three different days by quantification of deducted matrix blank samples *via* an internal calibration curve. The acceptable limit for accuracy and precision should be within ±20% and the RSD value must be less than 20%, respectively.

#### 2.5.5. Matrix effect and recovery of sample preparation

The matrix effect was evaluated by comparing the standard response of analytes in the matrix with the response in the solvent to describe the analyte efficiency. Meanwhile, the recovery assay was performed to evaluate the accuracy and precision of the sample preparation method. The recovery for the method was determined by the measurement of spiking samples after whole-sample preparation. In addition, internal standards were used to correct for analyte recovery across the sample preparation procedure in each individual sample. To develop a protocol for sample pre-preparation with high efficiency and improved recovery, different methods were compared.

#### 2.5.6. Stability

Stability experiments were carried out for 4 h, 12 h, and 7 days at 4 °C. Calculate the relative standard deviation of the response of the target substance in the sample with the newly prepared linear curve compared with pre-results, evaluate the sample stability with accuracy. The deviation should be within ±15–20%.

#### 2.5.7. Population analysis

A total of 122 urine samples were selected to be quantified for the concentration of AICAR through the validated method in this study. Additional populations such as different genders, different ages, and different periods of analytical persons were predefined in the study protocol. Fig. 6 shows the summary of the concentration of AICAR. Anonymous population analysis data are published with the informed consent of the parties.

### 2.6. Administration study

**Fig. 5:**
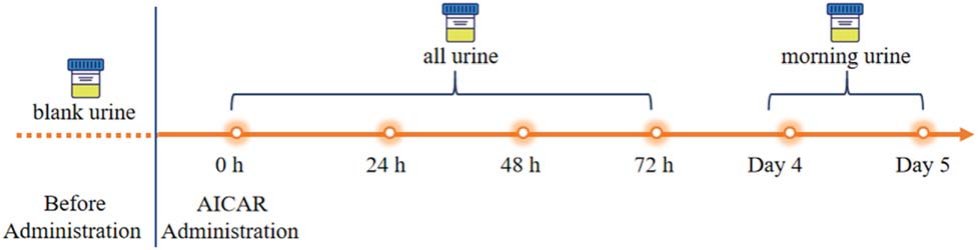
Scheme of the administration study.

To confirm the detection capabilities of the newly developed method, urine samples were obtained from four male volunteers who had orally taken AICAR and given informed consent (Fig. 5). Several blank urine samples were collected as baseline reference values, and after that, the AICAR drug trial was conducted. All urine samples were collected within 72 h of drug administration, and only morning urine was collected on the fourth and fifth days with an approximate volume. The samples collected from the AICAR subjects were divided into three groups: the blank samples before the test were designated as Group B, the samples collected within 0–16 hours after the administration were designated as Group A1, and the samples collected after 16 hours were designated as Group A2. The metabolic analysis of the sample testing data was then performed based on a machine learning model.

### 2.7. Statistical analysis

The entire raw dataset was processed rigorously using the MSDIA 4.9 instrument software. This involved critical preprocessing steps such as extracting peaks, correcting retention times, and aligning peaks to ensure data integrity. The key parameter settings ensured precision, with a retention time range spanning from 1 to 15 minutes, a retention time window of 0.1 minute, and a deviation tolerance of 10 PPM, while isotopic peaks were eliminated to minimize interference.

To identify metabolite structures, we leveraged precise mass number matching (within 10 ppm) and second-order spectrum matching (within 0.01 Da) techniques to search through renowned public databases such as HMDB, MassBank, and GNPS, as well as our proprietary BaiPu metabolite standard library. Prior to statistical analysis, ion peaks with over 50% missing values within a group were excluded to ensure data reliability. Subsequently, total peak area normalization was applied independently to positive and negative ion data. The normalized data were then consolidated, and the Python software was utilized for pattern recognition. Following unit variance (UV) scaling preprocessing, in-depth data analysis was performed.

The comprehensive multidimensional statistical analysis of metabolomics data was performed using Python (version 3.8.10). Differential metabolites between the two athlete groups, pre- and post-exercise, were identified using the Variable Importance in Projection (VIP) value from the Partial Least Squares-Discriminant Analysis (PLS-DA) model (VIP > 1) and the *p*-value (<0.05) from Student's *t*-test on normalized peak intensities. The Fold Change (FC) was calculated by comparing the mean normalized peak intensities between the two groups. Finally, the structural identification of these differential metabolites was accomplished through comprehensive searches in public databases such as HMDB, MassBank, and GNPS.

## 3 Result

### 3.1. Quantification method validation

#### 3.1.1. Selectivity

For selectivity testing, 27 different urine samples were collected and spiked with internal standard. These analyses were performed to evaluate the method's ability to distinguish target analytes from the endogenous matrix interference in samples. The selectivity was confirmed by comparing the chromatographic interference between samples and standards, and no interference was observed in all samples for product ions at the specified retention time (RT) (Fig. 3). Therefore, the method is selective and specific for the analysis of AICAR.

#### 3.1.2. Carry-over

No carry-over effect was observed by analyzing the reconstituted solvent injected after the highest calibration standard sample concentration (10 000 ng mL^−1^).

#### 3.1.3. Linearity range

Multiple standard samples were carefully prepared and used to construct calibration curves, allowing for the accurate and precise analysis of the relationship between concentration and response. The calibration curve exhibited excellent linearity across the range of 10–10,000 ng mL^−1^, with a 1/*X*^2^ weighting factor, meeting the specified criteria. The coefficient of determination (*R*^2^) exceeded 0.998 for all calibration curves during the analytical runs. Additionally, the mean equation derived from two non-zero calibration points was *y* = 0.00412*x* + 0.02149, demonstrating the robustness and reliability of the analytical method.

#### 3.1.4. Accuracy, precision, matrix effect and recovery

The accuracy and precision were determined for different spiked intra- and inter-day QC urine samples. The acceptance criteria results for the accuracy and precision are summarized in Table 1. The accuracy was calculated based on the established calibration curves and expressed as the relative value (in percent) = measured concentration value/actual concentration value × 100(%). The average accuracy after four batch QC tests was 104.3%, and the maximal validation accuracy was 112.8%, meeting the quantitative requirements (within ±20%) and demonstrating the quantitative possibilities of the established method. In addition, the results of within-day precision of the method were 1.0–15.6%, and similar results for the between-day precision were obtained, all less than 20%. To investigate the efficiency of the assay process, four replicates of the LLOQ and other QC samples were examined in order to evaluate the impact of matrix effect and extraction recovery on the analysis of AICAR. Overall, the differences of the matrix effects and extraction recovery for all the results were within 15%, which was fully compliant with the testing requirements (Table 1).

**Table 1:** Accuracy, precision, matrix effect and recovery of the method

QC level	Nominal concentration (ng mL^−1^)	Accuracy (%)	Precision (RSD%)		Matrix effect		Recovery	
			Intraday (*n* = 4)	Interday (*n* = 12)	(%)	Cv (*n* = 4)	(%)	Cv (*n* = 4)
LLOQ	10	112.8	15.6	16.3	88.9	4.9%	87.4	1.4%
LQC	100	104.0	2.6	2.5	101.8	3.4%	100.8	2.8%
MQC	1000	99.9	2.1	2.1	100.2	3.5%	104	1.4%
HQC	5000	100.4	1	1.3	103.6	2.2%	106.5	2.4%

**Fig. 6:**
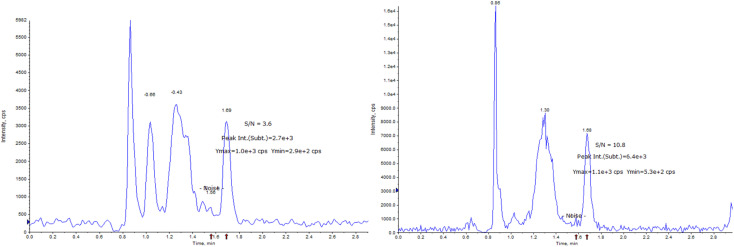
LOD (left), SNR = 3.6; LOQ (right), SNR = 10.8. SNR: signal-to-noise ratio.

The LOD and LOQ of the detection system were estimated by measuring the analytical signals of the three blank samples. As shown in Fig. 6, the LOD and LOQ of the method were 1 ng mL^−1^ and 10 ng mL^−1^, respectively, based on the signal-to-noise ratio (SNRLOD > 3, SNRLOQ > 10).

#### 3.1.5. Stability

Stability tests were conducted to evaluate the stability of the whole optimized detection process. The experiments were carried out for 4 h, 12 h, and 7 d at an instrument detection temperature of 4 °C (Table 2). It is worth noting that to ensure accuracy, samples at the LLOQ concentration (10 ng mL^−1^) need to be detected within 12 h because the analysis precision was just over 20% in 12 h. Aside from this, the AICAR analysis results were consistent at other QC levels (100, 1000, and 5000 ng mL^−1^) after 4 h, 12 h, and 7 days stored at detected phenomenon. After conducting stability and precision tests at the temperature of conventional detection, it was found that concentrations ranging from 100 to 5000 ng mL^−1^ can be stored for a week or even longer while still yielding relatively accurate results. However, for concentrations below the lower limit of quantification (LLOQ), measurements should be completed as soon as possible within 12 hours to avoid potential errors caused by degradation or other factors.

**Table 2:** Stability test for AICAR at different concentrations

Period	QC level (ng mL^−1^)	Accuracy^a^ (%)	Precision^a^ (*n* = 8, %)
0 h	10	112.8	15.6
	100	104	2.6
	1000	99.9	2.1
	5000	100.4	1
4 h	10	119.4	16.2
	100	102.8	4.5
	1000	99.7	2.6
	5000	99.7	2.6
12 h	10	116.4	23.9
	100	102.9	3.6
	1000	101	2.4
	5000	99	2.1
7 d	10	94.8	26.5
	100	102.1	8.2
	1000	100	2.6
	5000	100	1

a Accuracy and precision of quality control (QC) level should be within ±15–20% (20% only for the lowest concentration point of the standard).

### 3.2. Population quantitative analysis

The protocol of this study was approved by the ethics committee of Shanghai University of Sport, China. Ethics no 102772023RT110.

#### 3.2.1. Normal people

The level of endogenous AICAR is important in sports.^19^ Meanwhile, since AICAR can participate in the process of energy metabolism, the basic metabolic rate is different, and the metabolic ability is different between people of different sexes, races, and types of exercise.^20^ The endogenous AICAR levels required to participate in mediating metabolism are different, and the concentration differences are significant. In previous work, among the detection of 12377 North American athletes' urine, the mean concentration of AICAR was 647 ± 365 ng mL^−1^.^21^ Urine samples of 290 Asian athletes were tested with an average AICAR concentration of 1528 ± 1165 ng mL^−1^.^22^

**Fig. 7:**
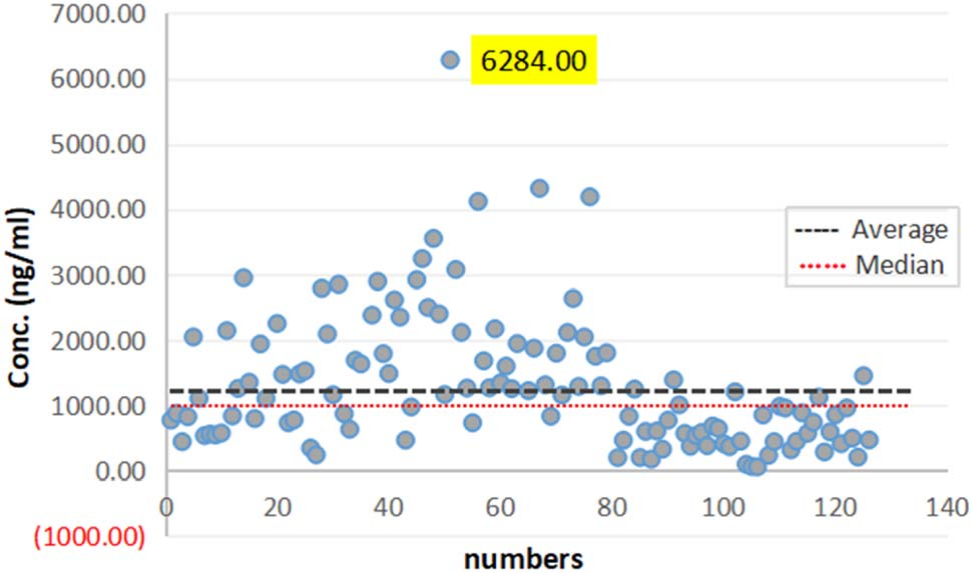
Population analysis of 122 urine samples.

In this work, samples form 122 participants were analyzed to assess the method accuracy (64 males and 58 females). Fig. 7 summarizes the AICAR concentrations. Based on the results, the population can be divided into three parts based on the concentration: the maximum concentration was 6284 ng mL^−1^ and the minimum was 53.2 ng mL^−1^. Meanwhile, the calculated average concentration of the samples was 1310.5 ng mL^−1^, and the standard deviation was 1031.4 ng mL^−1^. The findings indicated a notable disparity in the levels of concentrations between male and female participants with values of 1719.5 ± 1122.2 ng mL^−1^ and 859.1 ± 686.2 ng mL^−1^, respectively. This difference was deemed statistically significant, highlighting potential gender-related variations in the studied parameters, and the trends of the results were consistent with those in the literature.^22^

#### 3.2.2. People of Sanda training

**Fig. 8:**
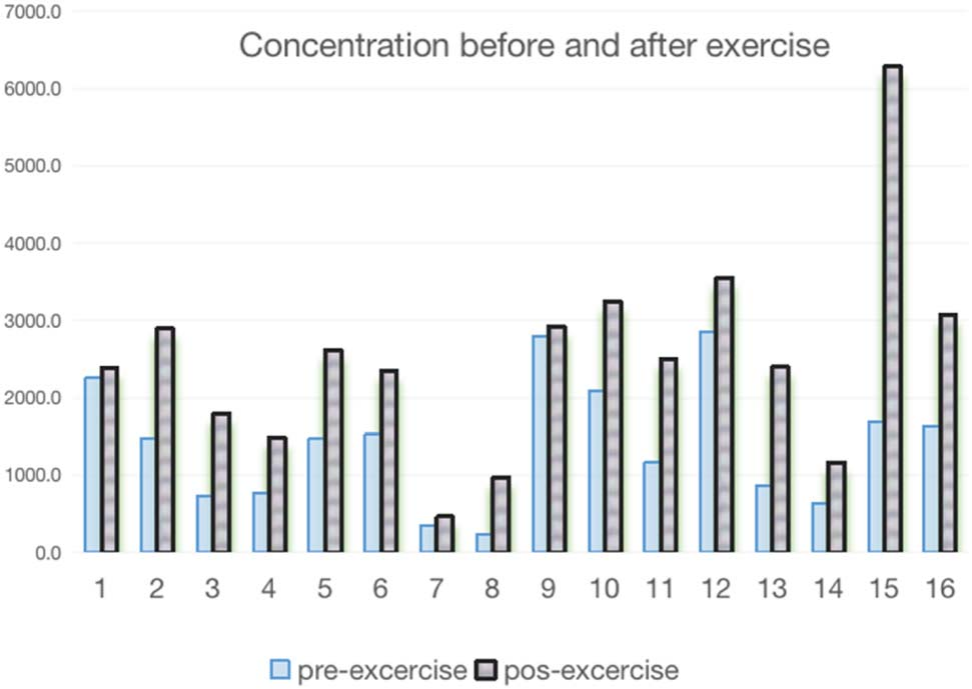
Concentration of AICAR before and after exercise.

Based on the premise of AICAR's involvement in exercise energy metabolism, in order to examine the changes of AICAR before and after exercise, 16 healthy students participating in boxing training were selected to be part of the study. Urine samples were collected before exercise on the first morning (labelled as Pre) and immediately after exercise (labelled as Post). The exercise regimen included 20 minutes of warm-up, 30 minutes of technical drills, 30 minutes of tactical training, and 40 minutes of actual combat. Totally, 32 urine samples were collected for pre-exercise and post-exercise. From the results in Fig. 8, we can clearly see that the concentration of AICAR increased significantly after exercise, promoting the activation of AMPK energy cycle metabolism.

#### 3.2.3. Administration study

**Fig. 9:**
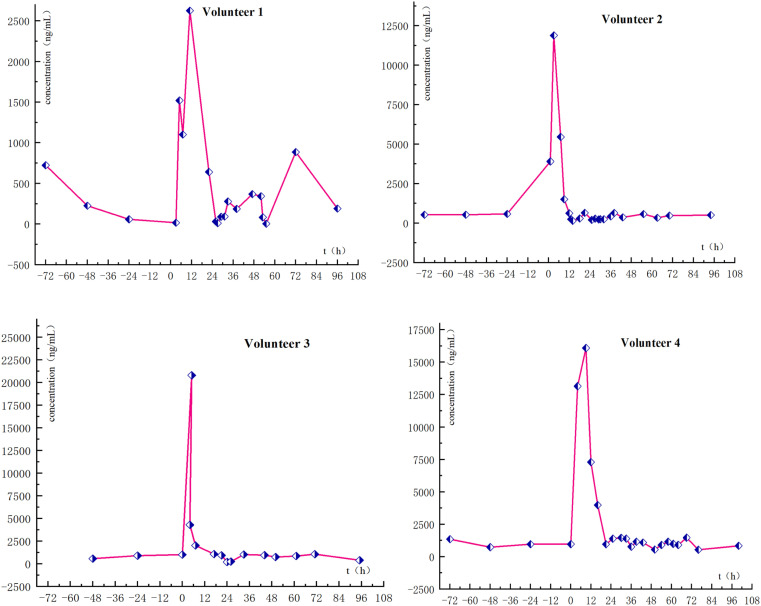
Concentration of AICAR after administration.

The results show that the concentration of AICAR significantly increases within 16 hours after taking the medicine, which can be used as a reference time point for drug metabolism clearance rate (Fig. 9).

### 3.3. Metabolomics profiling results

**Fig. 10:**
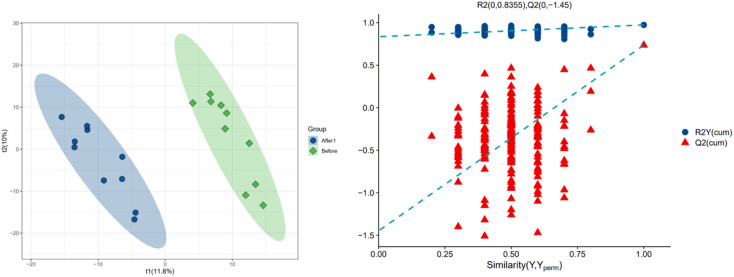
PLS-DA analysis of group A1 and B (left: PLS-DA; right: permutation test plot).

**Fig. 11:**
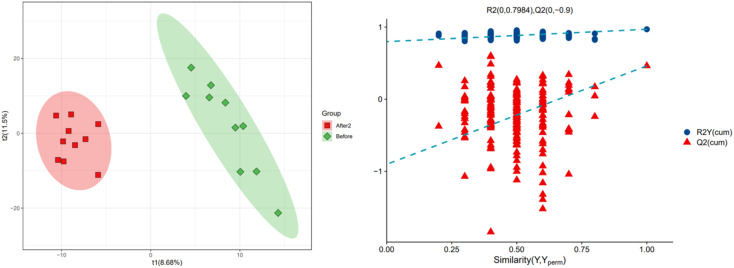
PLS-DA analysis of group A2 and B (left: PLS-DA; right: permutation test plot).

In order to further explore the impact on human body metabolites of AICAR administration, metabolomics analysis was performed to determine the different metabolites in urine and screen the biomarkers of consumption.^23^ In the positive and negative ion modes, 734 and 294 variables were extracted for data analysis, respectively, and over 100 differential metabolites were screened out (see ESI†). Due to the multidimensional characteristics of metabolomics data, it is necessary to “simplify and reduce the dimensionality” of multidimensional complex data on the basis of maximizing the retention of original information and establish reliable mathematical models to summarize the metabolic profile characteristics of research objects. Partial Least Squares Discriminant Analysis (PLS-DA) is a common multivariate statistical analysis method that can maximize the differences between groups according to predefined classifications. The PLS-DA results of urine samples in group A1 and group B are shown in Fig. 10 (left). It can be seen that the data within the groups are closely related, indicating good repeatability during sample testing, and the data between the groups are separated. This indicates that there are significant differences between the two sets of metabolomics data, and the metabolites in the urine of the AICAR-treated samples have undergone some changes compared to the pre-treated samples within 0–16 hours after treatment. The *R*^2^ and *Q*^2^ values are 0.974 and 0.736, respectively, indicating good stability and predictive ability of the model. Fig. 10 (right) shows the model validation of PLS-DA, which was tested 200 times with the permutation test. The horizontal axis represents the correlation between randomly grouped Y and original grouped Y, and the vertical axis represents the scores of *R*^2^ and *Q*^2^. The quality of the multivariate statistical analysis model should ultimately be based on the permutation test results. The *Q*^2^ value shown in the figure is −1.45, indicating that the model is very reliable and the PLS-DA model established from the experimental data has not been overfitted. The PLS-DA results of urine samples in group A2 and group B are shown in Fig. 11 (left). It can be seen that the data within the groups are closely related, while the data between the groups are separated. *R*^2^ and *Q*^2^ are 0.970 and 0.463, respectively, indicating that the model has good stability but general predictive ability. Fig. 11 (right) shows the model validation of PLS-DA. The figure shows that Q^2^ = −0.9, indicating that the model is very reliable and the PLS-DA model established from the experimental data has not been overfitted.

**Fig. 12:**
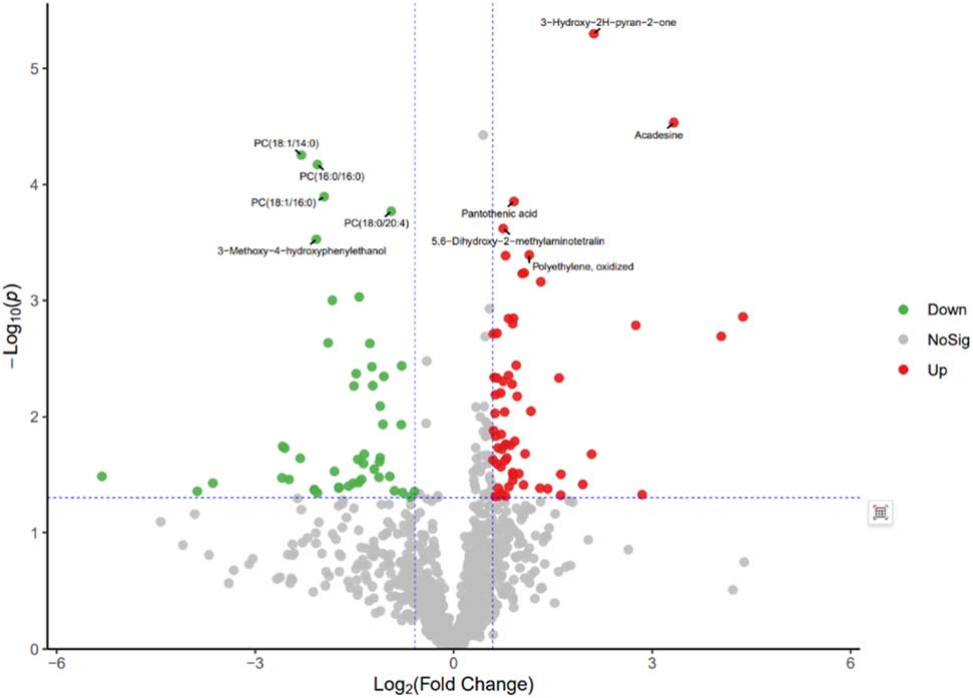
Volcano plot of differential metabolites between group A1 and group B.

The volcano plot integrates the univariate analysis method *t*-test, which can visually display the significance of metabolite changes between two groups of samples, thereby enabling the screening of potential marker metabolites. The volcano plot is shown in Fig. 12. The screening of differential metabolites is based on the criteria of VIP > 1, *p* < 0.05, and FC < 1/1.5 or FC > 1.5. Red represents metabolites up-regulated in group A1 compared to group B, and green represents metabolites down-regulated in group A1 compared to group B. The results show that there are a total of 143 significant metabolites, including 52 significantly down-regulated metabolites (47 in the positive ion mode and 5 in the negative ion mode) and 91 significantly up-regulated metabolites (58 in the positive ion mode and 33 in the negative ion mode). Lipid components after AICAR administration showed significant down-regulation compared to those before administration, including phosphatidylcholine (PC), phosphatidyl ethanolamine (PE), and lysophosphatidyl ethanolamine (LPE). PC, also known as lecithin, is abundantly present in the human brain, nervous system, circulatory system, immune system, and internal organs.^24^ PC is the most abundant phospholipid in the human body, particularly accounting for 70–80% of its dry weight in brain cells. It is one of the most important nutrients for the human body.^25,26^ PE, also known as cerebroside, is the second most abundant phospholipid in the body and mainly exists in the brain and spinal cord. In animals, it is generated through the pathway of CDP ethanolamine followed by phospholipase A, which then produces LPE. In addition, PC can also be converted from PE obtained through CDP ethanolamine or decarboxylation pathways. The types of PC involved in this study include PC(18:1/16:0), PC(16:0/18:0), and PC(16:0/16:0), PE type is PE18:0/20:4, and LPE type is 18:1.

**Fig. 13:**
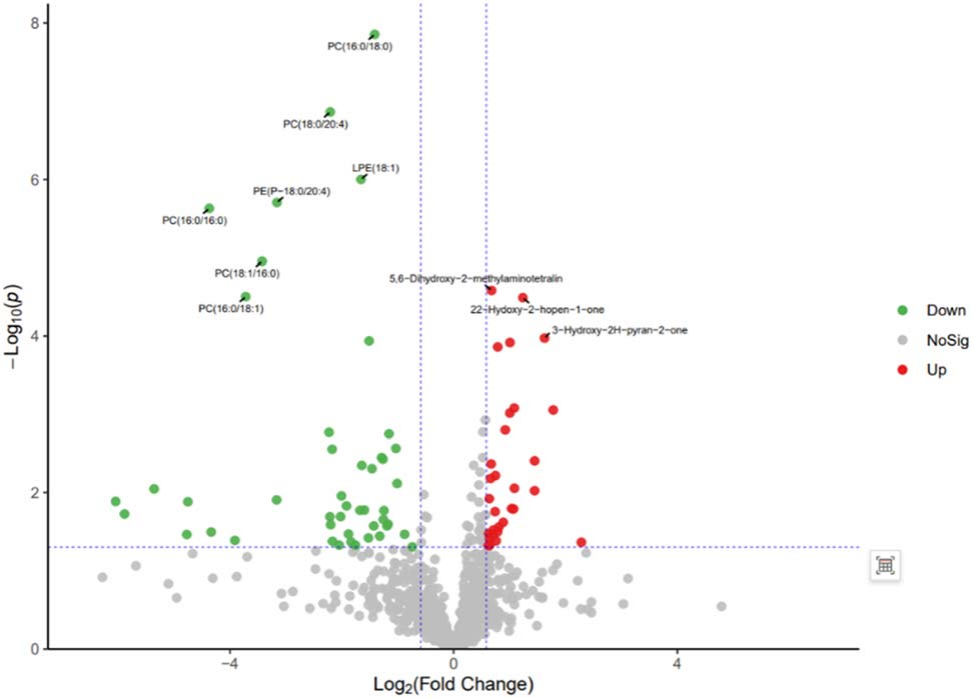
Volcano plot of differential metabolites between group A2 and group B.

The volcanic plot of group A2 compared with group B is shown in Fig. 13. Differential metabolites were screened based on VIP > 1, *p* < 0.05, FC < 1/1.5 or FC > 1.5. Red represents metabolites up-regulated in group A2 compared with group B, and green represents metabolites down-regulated in group A2 compared with group B. The results showed that there were 103 significant metabolites, of which 50 were significantly down-regulated metabolites, including 46 in the positive ion mode and 4 in the negative ion mode; 53 were significantly up-regulated metabolites, including 46 in the positive ion mode and 7 in the negative ion mode. By comparing with the volcanic plots of group A1 and group B, it was observed that the differential metabolites were significantly reduced, but the differential metabolites of phospholipids were significantly down-regulated.

**Fig. 14:**
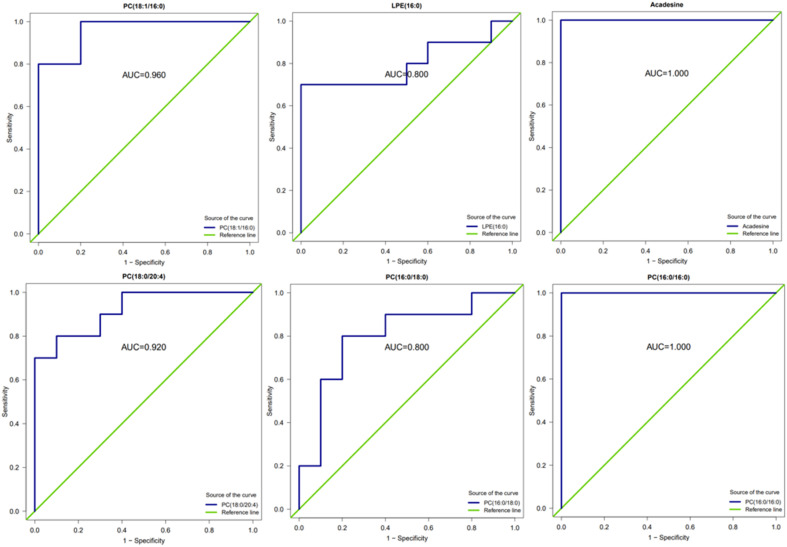
ROC diagram of related differential metabolites between group A1 and group B.

Receiver operating characteristic (ROC) curve analysis is a method that combines sensitivity and specificity to comprehensively evaluate the diagnostic accuracy or discrimination effect. The higher the area under the curve (AUC), the better the ability of such substances to distinguish between the comparison groups, which can be used as potential analytical markers. In this study, ROC analysis was performed on several substances with significant up- and down-regulation of differential metabolites between group A1 and group B (Fig. 14). The results showed that the area under the curve (AUC) was above 0.8 for all substances.

**Fig. 15:**
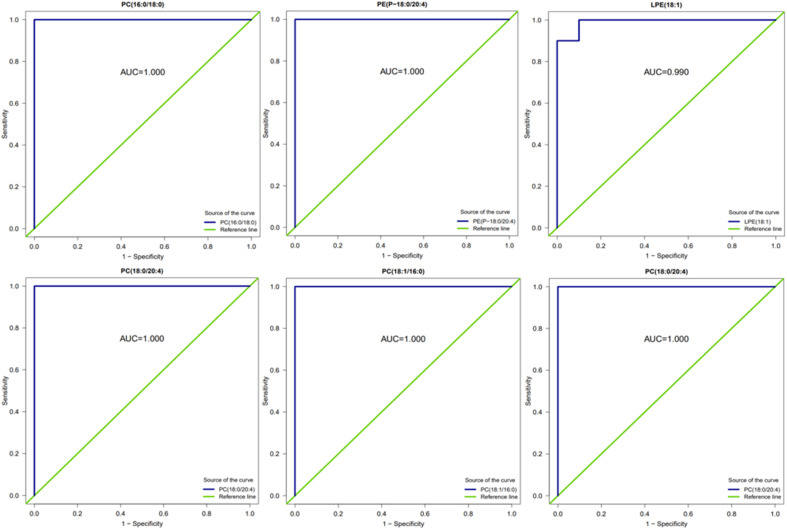
ROC diagram of related differential metabolites between group A2 and group B.

The ROC analysis was conducted for several substances with significant up- and down-regulation of differential metabolites in group A2 and group B, and the AUC results were almost 1. Fig. 15 shows that these substances can effectively distinguish between samples taken after 16 hours of AICAR administration and pre-administration samples. The results are also consistent with the ROC analysis of differential metabolites in group A1 and group B. Previous studies have shown that the AICAR affects the Kennedy pathway of PE synthesis. The incubation of AICAR with hepatocytes *in vitro* showed an increase in intracellular CDP ethanolamine,^27^ which was later confirmed by other researchers' experimental results.^28^ AICAR reduces the effect of glucose on fat production and fat decomposition. By culturing cells with AICAR and glucose separately and measuring the level of LPE, it was found that the level of LPE increased in glucose-treated cells, while the level of LPE decreased in AICAR-treated cells. PC, PE, and LPE can effectively distinguish between samples taken before and after administration of AICAR, and are expected to become potential biomarkers for detecting AICAR administration.

## 4 Conclusion

The quantitative detection method for the AICAR content in biological samples proposed in this study demonstrates extremely high sensitivity at a LLOQ concentration of 10 ng mL^−1^, which not only provides reliable technical support for the accurate detection of AICAR, but also demonstrates the profound strength and innovation ability of the research team in the field of biomedical analysis. The economic and environmental protection characteristics of this method indicate that in future applications, it can greatly reduce detection costs and environmental impact, contributing to the sustainable development of the biomedical industry.

In the study of human populations, we have revealed the differences in AICAR levels among normal people, professional athletes, and drug users. This finding not only has profound scientific value, but also provides important reference for the World Anti-Doping Agency (WADA) to develop more scientific and reasonable positive standards. By gaining a deeper understanding of the abuse of AICAR in the athlete population, we can more effectively maintain the fairness and purity of sports competitions and protect the physical and mental health of athletes.

In the study of drug metabolism, we found metabolites that were significantly up- and down-regulated after the administration of AICAR. Combining PLS-DA model validation and ROC curve analysis, it was found that there were significant changes in AICAR levels and other compounds such as PC-type PCs (18:1/16:0), PC(16:0/18:0), PC(16:0/16:0), PE-type PE18:0/20:4, and LPE-type 18:1. These findings enable us to not only better understand the efficacy and metabolic pathways of AICAR, but also successfully screen a series of phospholipids with significant discriminatory power as potential biomarkers. These biomarkers provide new ideas and methods for detecting AICAR administration.

In summary, the application of LC-MS/MS targeted quantitative and non-target omics analysis methods proposed in this study in the detection of AICAR provides powerful technical support for the fight against doping and promotes the fairness and healthy development of sports. In the future, we will further study and apply this method.

## Abbreviations

AICAR: 5-Amino-4-imidazolecarboxyamide ribonucleoside
AMPK: Adenosine 5′-monophosphate (AMP)-activated protein kinase
LC-MS/MS: Liquid chromatography-mass spectrometry
LOD: Limit of detection
LOQ: Limit of quantification
PLS-DA: Partial least squares discriminant analysis
ROC: Receiver operating characteristic
PC: Phosphatidylcholine
PE: Phosphatidyl ethanolamine
LPE: Lysophosphatidyl ethanolamine
WADA: World Anti-Doping Agency
PGC1α: Peroxisome proliferator activated receptor gamma coactivator 1 alpha
ZMP: AICAR-monophosphate
LKB1: Liver kinase B1
NTPs: Ribonucleoside triphosphates
MRM: Multiple reaction monitoring
ESI: Electrospray ionization
RT: Retention time
FA: Formic acid
UPLC: Ultra-performance liquid chromatography
QC: Quality control
VIP: Variable importance in the projection
FC: Fold change
AUC: Area under the curve
LLOQ: Lower limit of quantification
MQC: Middle quality control
LQC: Low quality control
HQC: High quality control

## Conflicts of interest

There are no conflicts to declare.

## Supplementary Material

RA-014-D4RA02878C-s001

## References

[cit1] Dite T., Langendorf C., Hoque A. (2018). *et al.*, AMP-activated protein kinase selectively inhibited by the type II inhibitor SBI-0206965. J. Biol. Chem..

[cit2] Tripathi V., Jaiswal P., Assaiya A. (2022). et al.. Curr. Cancer Drug Targets.

[cit3] Giri S., Rattan R., Haq E. (2006). *et al.*, AICAR inhibits adipocyte differentiation in 3T3L1 and restores metabolic alterations in diet-induced obesity mice model. Nutr. Metabol..

[cit4] Gao J., Xiong R., Xiong D. (2018). *et al.*, The Adenosine Monophosphate (AMP) Analog, 5-Aminoimidazole-4-Carboxamide Ribonucleotide (AICAR) Inhibits Hepatosteatosis and Liver Tumorigenesis in a High-Fat Diet Murine Model Treated with Diethylnitrosamine (DEN). Med. Sci. Mon..

[cit5] Krishnan U., Viswanathan P., Venkataraman A. (2023). AMPK activation by AICAR reduces diet induced fatty liver in C57BL/6 mice. Tissue Cell.

[cit6] Terai K., Hiramoto Y., Masaki M. (2005). *et al*, AMP-activated protein kinase protects cardiomyocytes against hypoxic injury through attenuation of endoplasmic reticulum stress. Mol. Cell. Biol..

[cit7] Samsonov M. V., Podkuychenko N. V., Khapchaev A. Y. (2021). et al.. Int. J. Mol. Sci..

[cit8] Wang L., Di L.-J. (2015). Wnt/β-catenin mediates AICAR effect to increase GATA3 expression and inhibit adipogenesis. J. Biol. Chem..

[cit9] Zhou W., Zhang J., Marcus A. I. (2014). LKB1 tumor suppressor: Therapeutic opportunities knock when LKB1 is inactivated. Genes Dis..

[cit10] Villa E., Ali E. S., Sahu U., Ben-Sahra I. (2019). Cancer cells tune the signaling pathways to empower de novo synthesis of nucleotides. Cancers.

[cit11] Gonzalez-Franquesa A., Peijs L., Cervone D. T. (2021). et al.. Proteomes.

[cit12] Thevis M., Thomas A., Kohler M. (2009). *et al.*, Emerging drugs: mechanism of action, mass spectrometry and doping control analysis. J. Mass Spectrom..

[cit13] World Anti-Doping Agency (WADA) , The world anti-doping code: the 2009 prohibited list international standard, http://www.wada-ama.org/rtecontent/document/2009_List_En.pdf, 2009, accessed April 22, 2021

[cit14] D'Atri V., Fekete S., Clarke A., Veuthey J.-L., Guillarme D. (2019). Recent advances in chromatography for pharmaceutical analysis. Anal. Chem..

[cit15] Thomas A., Beuck S., Eickhoff J. C., Guddat S., Krug O., Kamber M., Schänzer W., Thevis M. (2010). Quantification of urinary AICAR concentrations as a matter of doping controls. Anal. Bioanal. Chem..

[cit16] Guddat S., Solymos E., Orlovius A. (2011). et al.. Drug Test. Anal..

[cit17] Dmitrieva E. V., Temerdashev A. Z., Azaryan A. A., Gashimova E. M. (2019). Application of solid-phase extraction for the quantification of urinary AICAR by ultra-high performance liquid chromatography–tandem mass-spectrometry. J. Anal. Chem..

[cit18] Kohyama N., Hayashi T., Yamamoto Y. (2005). Synthesis of a methylene analog of 5-Amino-1-β-D-ribofuranosylimidazole-4-carboxamide monophosphate (ZMP). Biosci. Biotechnol. Biochem..

[cit19] WADA Laboratory Expert Group , WADA Technical Document TD2015IDCR, 2015, http://www.wada-ama.org/sites/default/files/resources/files/td2015idcr_-_eng.pdf

[cit20] Houghton E. and Maynard S., Some aspects of doping and medication control in equine sports, in, Handbook of Experimental Pharmacology, ed. D. Thieme and P. Hemmersbach, Springer, Berlin, Heidelberg, 195, 2010, pp. 369–409 10.1007/978-3-540-79088-4_17 20020374

[cit21] Sobolevsky T., Ahrens B. (2019). Urinary concentrations of AICAR and mannitol in athlete population. Drug Test. Anal..

[cit22] Hong Y., Xu Y. (2016). The detection method establishment and application for LC-MS/MS. Chin. J. Sports Med..

[cit23] Hashempour S., Shahabadi N., Adewoye A. (2020). *et al.*, Binding Studies of AICAR and Human Serum Albumin by Spectroscopic, Theoretical, and Computational Methodologies. Molecules.

[cit24] Cole L. K., Vance J. E., Vance D. E. (2012). Phosphatidylcholine biosynthesis and lipoprotein metabolism. Biochim. Biophys. Acta.

[cit25] van der Veen J. N., Kennelly J. P., Wan S. (2017). *et al.*, The critical role of phosphatidylcholine and phosphatidylethanolamine metabolism in health and disease. Biochim. Biophys. Acta Biomembr..

[cit26] Calzada E., Onguka O., Claypool S. M. (2016). Phosphatidylethanolamine Metabolism in Health and Disease. Int. Rev. Cell Mol. Biol..

[cit27] Houweling M., Klein W., Geelen M. J. (2002). Regulation of phosphatidylcholine and phosphatidylethanolamine synthesis in rat hepatocytes by 5-aminoimidazole-4-carboxamide ribonucleoside (AICAR). Biochem. J..

[cit28] ElAzzouny M. A., Evans C. R., Burant C. F., Kennedy R. T. (2015). Metabolomics Analysis Reveals that AICAR Affects Glycerolipid, Ceramide and Nucleotide Synthesis Pathways in INS-1 Cells. PLoS One.

